# Infection et descellement d’une prothèse totale de la hanche par *listeria monocytogenes*

**DOI:** 10.11604/pamj.2018.30.18.14016

**Published:** 2018-05-09

**Authors:** Abdelhafid El Marfi, Abdelghani Elayoubi, Mohamed El Idrissi, Mohamed Shimi, Abdelhalim El Ibrahimi, Abdelmajid Elmrini, Hassan Fokiladeh

**Affiliations:** 1 Service de Chirurgie Ostéo-articulaire B4, CHU Hassan II, Université Sidi Mohammed Ben Abdellah, 3000 Fès, Maroc; 2 Service de Chirurgie Orthopédique et Traumatologie, Centre Hospitalier René Dubos, 95300 Pontoise, France

**Keywords:** Listeria, prothèse totale de la hanche, descellement, antibiothérapie, reprise, Listeria, total hip prosthesis, detachment, antibiotics, recovery

## Abstract

Le recours aux prothèses articulaires est de plus en plus fréquent dans notre pratique courante. Une des complications les plus redoutées est l’infection, le plus souvent due à des germes de la flore cutanée. Listeria monocytogenes étant rarement à l’origine d’une telle infection, nous rapportons le cas d’un patient porteur d’une prothèse de hanche infectée par ce germe: l’évolution a été favorable après antibiothérapie et changement de prothèse en un seul temps.

## Abstract (en)

Joint prostheses are increasingly used in our current practice. Infection is one of the most dreaded complications; it is most often due to germs of the skin flora. Listeria monocytogenes rarely causes this infection. We here report the case of a patient with hip prosthesis infected by this germ. Favorable outcome was obtained by antibiotic therapy and single-stage prosthesis replacement.

## Introduction

Listeria est une cause inhabituelle d’infection des prothèses articulaires et leurs descellements. On rapporte un cas d’un descellement septique d’une prothèse totale de la hanche (PTH) à listeria.

## Patient et observation

**Figure 1:**
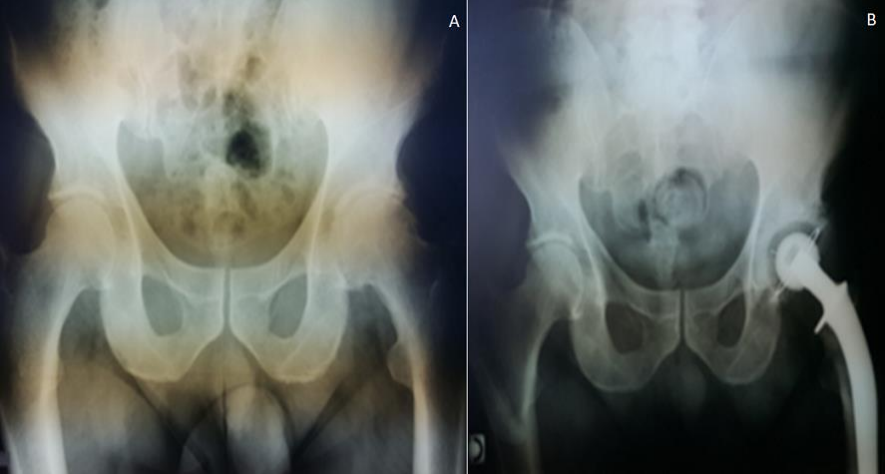
A) coxarthrose gauche; B) PTH cimentée radiographie du contrôle

**Figure 2:**
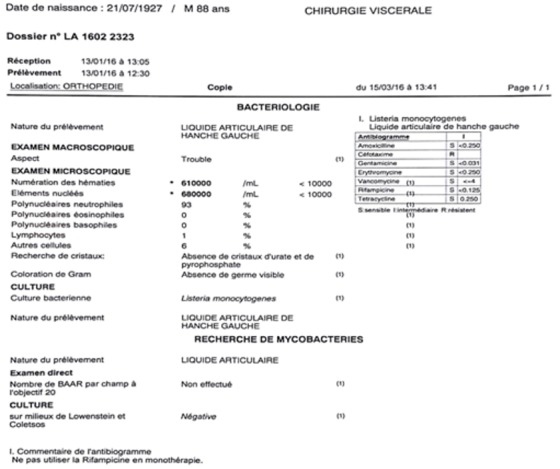
Résultats bactériologiques de la ponction de la hanche

**Figure 3:**
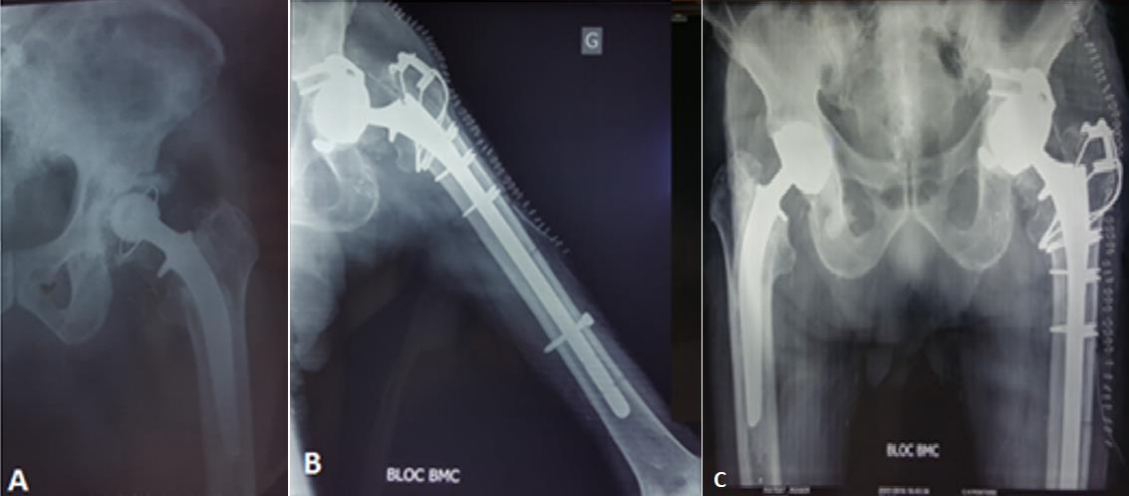
A) descellement bipolaire de la PTH; (B, C) contrôle radiologique de la reprise par une PTH double mobilité avec anneau de soutien de kerboull et une tige longue verrouillée

**Figure 4:**
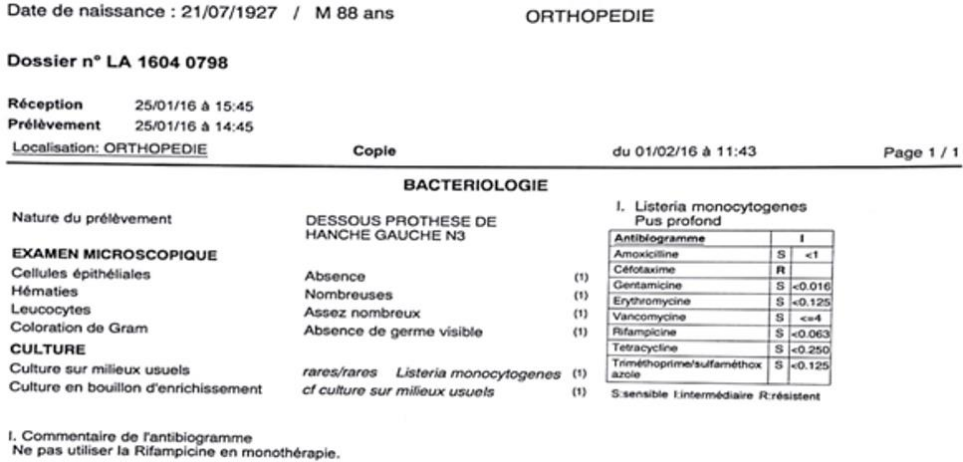
Résultats bactériologiques des prélèvements per-opératoires

Il s’agit d’un patient P.R âgé de 88 ans, ayant comme antécédents une hypertension artérielle sous traitement, un accident vasculaire cérébral ischémique sans séquelles, porteur de PTH gauche sur coxarthrose depuis 20 ans (Figure 1), PTH droite posée il y a 8 ans, patient autonome , marche avec canne , vit seul avec des aides à domicile. Le patient lors de son séjour au service de gériatrie aigue pour bilan de chute à répétions. Il a présenté des douleurs de la hanche gauche avec un syndrome inflammatoire biologique. L’examen clinique ne trouve pas de fistule, ni de signes inflammatoires locaux, la mobilité de la hanche a été douloureuse avec un flexum de 10° et raccourcissement du membre inférieur gauche de presque 1cm. Les radiographies du bassin et de la hanche gauche face et profil ont objectivé un descellement bipolaire de la PTH. Une ponction de la hanche a été réalisée avant d’envisager une révision chirurgicale ramenant un liquide trouble dont l’étude bactériologique a révélée une listeria monocytogenes (Figure 2). Le patient a été programmé après pour reprise avec lavage et changement de prothèse en un seul temps (Figure 3). Les prélèvements réalisés au bloc opératoire ont révélé la listeria monocytogenes (Figure 4). Le patient a été mis sous Antibiotique à base de Gentamycine à dose de 5mg/Kg/jour pendant 5 jours associé à l’Amoxcilline 2gx3/jour en intraveineux pendant 7jours, puis relais par voie orale pendant 3 mois. Le patient a été vu à 1mois puis à 2 mois avec une amélioration clinique et reprise de la marche avec un déambulateur, cicatrice acquise et syndrome inflammatoire qui a régressé. Au dernier recul de 3 mois le patient a pu reprendre son mode vie antérieur.

## Discussion

L’infection de prothèse totale de hanche (IPTH) est une complication grave mettant en jeu le pronostic vital et fonctionnel. Sa fréquence est de 1 à 2%. Son traitement repose sur une stratégie médicochirurgicale définie au cours de RCP (réunion de concertation pluridisciplinaire). Son but est double: guérir l’infection et restaurer la fonction avec le minimum de séquelles. Le diagnostic bactériologique est une étape fondamentale. Il est réalisé sur de multiples prélèvements per opératoires faits suivant une méthodologie rigoureuse. Les prélèvements doivent être confiés à un laboratoire capable de traiter les prélèvements solides et de cultiver des bactéries exigeantes difficilement cultivables, surtout dans les infections chroniques.

Le traitement chirurgical est indispensable à la guérison d’une IPTH. Dans les infections aiguës, une excision-lavage est indiquée en urgence. Tout retard en compromet le résultat. Dans les infections chroniques, l’ablation de la prothèse et de tout le matériel étranger est la base. C’est une intervention souvent complexe et toujours lourde. La repose d’une nouvelle prothèse dans le même temps chirurgical ou lors d’un deuxième temps reste controversée, aucune étude ne permettant de conclure [1-3]. Toutes sortes d’espèces bactériennes peuvent être rencontrées cependant les bactéries à Gram positif restent les plus fréquents 80% des cas [4]. Le choix de l’antibiotique dépend de plusieurs facteurs (sensibilité de germes isolés, diffusion osseuse, allergie, terrain…) [5], l’association de plusieurs antibiotiques est recommandé par plusieurs auteurs [6], pour une durée suffisante pour la cicatrisation des tissu osseux [4,7]. La *listeria monocytogenes* est un bâtonnet Gram positif non mobile et non sporulant. Une immunité cellulaire défectueuse est un facteur de risque de listériose invasive. Des auteurs ont rapporté des cas d’infection de prothèse de genou à listeria chez des patients atteints de PR [8], aussi on a monté un cas d’arthrite du genou chez un patient receveur d’une greffe rénale [9].

Dans notre cas l’isolement de *listeria* a été fait sur tous les prélèvements effectués. La *listeria* n’est pas un germe nosocomial. Elle est très sensible aux antibiotiques, sa sensibilité est presque constante à l’amoxicilline. Ce travail est le premier décrivant un descellement septique d’une PTH à *listeria monocytogenes*.

## Conclusion

L’infection isolée d’une prothèse de la hanche par la listeria est exceptionnelle, dont la prise en charge rejoints celle des infections des prothèses qui répond à des règles précises après un staff RCP et souvent dans des structures hospitalières habituées à ce genre de patients.

## Conflits d’intérêts

Les auteurs ne déclarent aucun conflit d’intérêts.
